# Spontaneous Bacterial Peritonitis due to* Lactobacillus paracasei* in Cirrhosis

**DOI:** 10.1155/2018/5714053

**Published:** 2018-03-01

**Authors:** Emily Harding-Theobald, Bharat Maraj

**Affiliations:** Department of Medicine, University of California, San Francisco Medical Center, San Francisco, CA, USA

## Abstract

*Lactobacillus* species colonize the human gastrointestinal tract and are rarely pathogenic. We present a case involving a cirrhotic patient who presented with sepsis and was found to have peritoneal cultures demonstrating* Lactobacillus* as the sole pathogen concerning for spontaneous bacterial peritonitis. Treatment was achieved with high-dose penicillin and clindamycin but the patient developed hepatorenal syndrome and died from acute renal failure. Intra-abdominal* Lactobacillus* infections are typically seen in patients undergoing peritoneal dialysis or who have recently had bowel perforation. There are few case reports of spontaneous* Lactobacillus* peritonitis in patients with cirrhosis. Our case report addresses the challenges of* Lactobacillus* treatment and suggests antibiotic coverage of commensal organisms in patients who do not improve with standard management.

## 1. Introduction

*Lactobacillus* species are facultative anaerobic gram-positive bacilli that are part of normal human gastrointestinal flora and are seldom pathogenic [1]. We present a rare case of spontaneous* Lactobacillus* peritonitis in a cirrhotic patient which illustrates the importance of early microbiologic studies in patients with spontaneous bacterial peritonitis unresponsive to empiric antibiotic therapy.

## 2. Case Presentation

A 73-year-old male with alcoholic cirrhosis complicated by prior spontaneous bacterial peritonitis on ciprofloxacin prophylaxis presented to the emergency department with fever, abdominal pain, and nausea. On admission he had normal vital signs. His abdomen was distended and diffusely tender with a positive fluid wave. Laboratory results revealed a leukocytosis to 18,200 cells/mL, stable liver function tests, and a creatinine of 1.8 mg/dL. His Model for End-Stage Liver Disease (MELD) score was 18 on initial presentation. Paracentesis revealed hazy peritoneal fluid with 2,590 white blood cells of which 2,434 were neutrophils. He was started on vancomycin and ertapenem for sepsis secondary to spontaneous bacterial peritonitis but progressively worsened clinically. On day 4 of hospitalization, the patient became tachycardic and hypotensive with a leukocytosis of 29,900 cells/mL. Repeat paracentesis revealed 2,670 neutrophils. A CT scan of the abdomen did not show evidence of focal infection or gastrointestinal perforation. Peritoneal fluid cultures revealed* Lactobacillus paracasei* as the sole pathogen isolated from both aerobic and anaerobic plates. The isolate was resistant to carbapenems and sensitive to clindamycin and penicillin. The patient was switched to clindamycin and high-dose intravenous penicillin (2 million units every 4 hours) for* Lactobacillus* coverage. Despite a rapid improvement in his peripheral leukocytosis and hemodynamic measures, the patient's creatinine continued to rise rapidly. He ultimately developed type 2 hepatorenal syndrome with anuric renal failure. He was transitioned to comfort care and died of multiorgan failure.

## 3. Discussion

*Lactobacillus* species are rarely implicated in spontaneous abdominal infections. Previous case reports describe secondary* Lactobacillus *peritonitis in the setting of peritoneal dialysis, recent intra-abdominal surgery, or gastrointestinal perforation [2–6]. Spontaneous* Lactobacillus* peritonitis in the absence of these risk factors is exceptionally rare and has exclusively been reported in severe decompensated cirrhosis with ascites [2, 7]. Given the impairment of intestinal barrier function and relative immunodeficiency created by cirrhosis, spontaneous* Lactobacillus *peritonitis most likely represents seeding of ascites via transmural migration across the bowel wall. Importantly,* Lactobacillus* is unusual in that it is intrinsically resistant to vancomycin and commonly resistant to cephalosporins [1]. As a result, infection with* Lactobacillus* is unlikely to respond to empiric antibiotic choices for spontaneous bacterial peritonitis. The most widely studied treatment for* Lactobacillus* peritonitis is high-dose intravenous penicillin (12–20 million units per day) in combination with aminoglycoside or clindamycin [8].

The clinical significance of* Lactobacillus* spp. cultured from sterile sites has been widely debated, and certainly not all cultures are indicative of true infection [9]. When* Lactobacillus* is cultured in the setting of abdominal viscus perforation, cultures are typically polymicrobial [2]. Our patient had a large neutrophilic predominance in his ascitic fluid with cultures growing only a single* Lactobacillus* isolate.* Lactobacillus paracasei* was identified in our microbiology laboratory with a gram stain revealing slender gram-positive rods in chains and after forming alpha-hemolytic colonies on blood agar which were hydrogen sulfide and catalase negative. The frequency of this clinical entity may be underrecognized, as* Lactobacilli *exhibit variable gram stain morphology and minimal growth on typical media [10]. Our patient responded dramatically to appropriate therapy with both clinical and symptomatic improvement. It may therefore be appropriate to broaden empiric treatment to cover* Lactobacillus* in patients not responding to typical gram-negative coverage. While clinically significant infection with* Lactobacillus *is rare, this case underlines the importance of early recognition and treatment of atypical pathogens in patients presenting with spontaneous bacterial peritonitis and cirrhosis.
